# The Effectiveness of Professional Development in the Self-Efficacy of In-Service Teachers in STEM Education: A Meta-Analysis

**DOI:** 10.3390/bs15101364

**Published:** 2025-10-06

**Authors:** Jiao Liu, Ke Wang, Zilong Pan

**Affiliations:** 1 Independent Researcher, Shanghai 200062, China; 2 School of Teaching and Learning, Sam Houston State University, Huntsville, TX 77341, USA; 3 Teaching Learning and Technology, Lehigh University, Bethlehem, PA 18015, USA

**Keywords:** in-service STEM teacher, meta-analysis, professional development, self-efficacy

## Abstract

This meta-analysis reports on the effect of professional development (PD) on K-12 in-service STEM teachers’ self-efficacy. There were 18 selected empirical studies in this study. Overall, PD had a modest positive effect on self-efficacy (Hedges’*g* = 0.551, 95% CI [0.285, 0.704], SE = 0.107) under the random-effects model. Furthermore, the findings show that (1) participant size of PD significantly contributed to the effect size of PD; (2) training hours of PD significantly contributed to the effect size of PD; (3) PD using the Teaching Efficacy Belief Instrument or other scales of self-efficacy showed larger significant effect sizes than PD using the Teachers’ Sense of Efficacy Scale. This study offers insights into the design of effective PD to improve STEM teachers’ self-efficacy.

## 1. Introduction

The development of information technology in the 21st century has brought about substantial changes in every aspect of human social life. Education, especially STEM education, is being pushed to fit the demands of human development. Nowadays, the need for STEM talents is huge. The cultivation of STEM talents is urgent, so the topic of STEM education has quickly become a significant area of interest (Y. Li, 2018; Y. Li et al., 2020; English, 2016). Many countries have designed plans for STEM education to face the urgent need for STEM talent. In the USA, the Committee on STEM Education released the STEM Education Strategic plan, *Charting a Course for Success: America’s Strategy for STEM Education* (The Committee on STEM Education, 2018). The Chinese Government issued the *China STEM Education 2029 Innovation Action Plan* in 2018. Australian Education Ministers issued the National STEM School Education Strategy 2016–2026 in 2015 (Education Council, 2015). Although investment in STEM education is increasing, the shortage of certified teachers in STEM is serious in the USA and other countries.

Meanwhile, all stakeholders are making great efforts to pursue solutions to the problem of the STEM teacher shortage. With the development of STEM education, the requirements for STEM teachers are increasing so that teachers can implement new curriculum standards. In particular, during the year 2020 (the year of the COVID-19 pandemic), the teaching requirements for STEM teachers significantly increased. Teachers had to transition to an online format from a traditional face-to-face one. However, a large number of teachers who graduated before 2015 were underprepared to teach in an online environment. Therefore, it is urgent to equip those in-service teachers with all kinds of teaching skills in the online teaching environment and after the COVID-19 period.

Research shows that there is a negative relationship between teaching requirements and in-service teachers’ self-efficacy. For example, the increase in online teaching requirements caused by COVID-19 contributes to the decrease in in-service teachers’ self-efficacy (Ma et al., 2021; Pressley & Ha, 2021). Online teaching created more challenges for in-service STEM teachers, who often do manipulatives and experiments. During the pandemic, almost all teachers had to transition from face-to-face teaching to virtual online teaching. Consequently, teachers need to learn all kinds of technological skills to deliver content successfully. Most importantly, teachers are facing the challenge of improving students’ learning results after the COVID-19 period. During the COVID-19 period, many students lagged far behind the requirements of the common core curriculum standards. Because of the need of improving teachers’ self-efficacy in instruction in the online learning environment and the possibility of STEM teachers’ leaving their jobs due to low self-efficacy (Aloe et al., 2014; Shoji et al., 2016), the need for professional development (PD) focused on improving in-service STEM teachers’ self-efficacy seem to be critically important (Hartshorne et al., 2020; Trikoilis & Papanastasiou, 2020).

Although there are some existing meta-analyses, scoping reviews, and systematic reviews about PD or self-efficacy (Egert et al., 2018; Gesel et al., 2020; Lynch et al., 2019), limited literature review focuses on PD and in-service STEM teachers’ self-efficacy. For example, Egert et al. (2018) analyzed the impact of in-service PD programs for early childhood teachers on standardized quality ratings and child outcomes. This study focused on early childhood teachers’ PD rather than K-12 STEM teachers. Also, this study did not evaluate the impact of PD on teacher self-efficacy. Gesel et al. (2020) evaluated the impact of PD on teachers’ knowledge, skills, and self-efficacy by synthesizing studies focusing on PD’s effect on in-service and preservice teachers’ knowledge, skills, and self-efficacy. Meanwhile, Gesel and his colleagues selected studies that targeted curriculum-based measurement and data-based decision-making PD. Although Lynch et al. (2019) targeted STEM PD and curriculum programs, this study examined the relationships between content, activities, or formats of PD and student outcomes. However, in the current study, we evaluated the impact of PD on STEM in-service teachers’ self-efficacy by analyzing the selected studies. The targeted studies focused on in-service teachers’ PD rather than preservice teachers. In addition, we analyzed the moderation effects of the features of PD on improving STEM teachers’ self-efficacy. It is quite helpful for teacher educators to have a clearer picture of effective PD.

### 1.1. Professional Development and Features of Effective PD

PD is defined as a series of designed activities that aim to improve teachers’ and administrators’ abilities to improve their practice and performance and to satisfy external demands (Elmore, 2004). It is commonly acknowledged that PD is a useful approach to improving teaching abilities (You et al., 2025; Zhou et al., 2023). Although PD programs (PDs) have been used to improve teachers’ professional knowledge, the effects of PD on teachers’ abilities are not always significant. As Villegas-Reimers (2003) stated, a well-designed, supported, and funded PD is the foundation of the effectiveness of PD. The features of the effective PD must be identified. Many scholars have been working on the features of effective PD. For example, Garet et al. (2001) concluded that effective teacher PD must include five aspects: (a) extended time and duration for learning to take place over many hours and days, (b) a focus on content knowledge or pedagogical content knowledge, (c) active learning related to teaching, (d) collective participation in teams, and (e) coherence with teachers’ PD experiences and alignment to standards. Based on Garet et al. (2001), Desimone (2009) developed a core conceptual framework for studying the effect of PD on teachers and students. The core feature of PD includes content focus, active learning, coherence, duration, and collective participation. Later, the National Academies of Sciences, Engineering, and Medicine (2015) adopted this framework to report on Science Teachers’ Learning. Meanwhile, Little and Paul (2009) proposed a comprehensive framework for effective PDs. It includes all stakeholders: (a) coherence, (b) climate, (c) instructional strategies, (d) participant engagement, (e) logistical considerations for participant learning, and (f) assessment and feedback. In addition, Darling-Hammond et al. (2017) argued that effective PD must (a) focus on content, (b) incorporate active learning, (c) support collaboration among participations, (d) use models of effective teaching practice, (e) provide professional coaching and support, (f) offer valuable feedback and reflection, and (g) sustain a reasonable duration. In sum, the above common features of effective PD include content, format, and training hours. In this study, we will adopt Desimone’s (2009) framework to analyze the effect of PD. We will describe them in the following.

### 1.2. Content of Effective Professional Development

PD can have different foci: subject content knowledge, pedagogical knowledge, curriculum knowledge, educational technology knowledge, and others (Elmore, 2004). Supovitz and Turner (2000) examined teachers’ evaluation of PD and found that content knowledge is the first need for teachers to implement inquiry-based teaching. Kennedy (1998) found that the significant differences among PDs were the content that was provided to teachers, and not the program forms or structures.

Loucks-Horsley and Bybee (1998) focused on science teachers’ PD and argued that effective PDs should include content knowledge that teachers really need, student learning, forms of instruction and assessment, and education reform. In addition, Darling-Hammond et al. (2009) found that effective PDs, including both content and pedagogy, positively affect teachers’ influence over student learning. Furthermore, Kanter and Konstantopoulos (2010) found that PDs focusing on a project-based science curriculum significantly influenced urban teachers’ content and pedagogical content knowledge. Therefore, effective PD can focus on increasing teachers’ knowledge and skills for lesson implementation (Jeanpierre et al., 2005).

Specifically, content knowledge could include a single discipline or multiple disciplines of STEM. For example, PDs focusing on STEM could include a single discipline (i.e., mathematics, science, technology, engineering) or a combination of two or three subjects (i.e., science and mathematics; technology, engineering, and science, etc.). Chen et al. (2023) stated that the integration of computer science knowledge into K-12 education is essential for teachers to enhance students’ computational thinking.

### 1.3. Format of Professional Development

There are three common models of in-service teachers’ PD: traditional, horizontal learning, and online (Gaudin & Chalies, 2015). Traditional PD is most prevalent and used for single- or multiple-day workshops focusing on information delivery (Avery, 2010). This short-term PD might have a short duration and lack the depth of knowledge required for it to be effective (Shields et al., 1998). However, horizontal learning PD focuses on peer learning and professional-network learning. One of the significant strengths is durative. This continuing PD is essential for teachers to develop their procedural and declarative knowledge (Knight, 2002). Finally, the online learning format is relatively new, and limited studies have examined its effect.

On the other hand, Garet et al. (2001) classified PD into two categories: traditional and reform. Traditional formats include workshops, conferences, and courses. Regularly, a leader or leaders with special expertise hold these PDs outside of teachers’ schools. However, the reform formats include study groups or mentoring and coaching. These PDs are held during the regular school day, focus on reform activities (mentoring in the whole process of lesson plan, implementation, and reflection), make connections with their teaching concerns, and are more responsive to teachers’ learning, teaching, needs, and goals (Garet et al., 2001).

### 1.4. Training Time

Although there is no exact duration for effective PD, it is commonly acknowledged that time is an important factor in highly qualified PDs. Research shows that short-term PD is not effective and that most teachers will revert to the previous status. It is essential to realize that teachers need more time to facilitate a change in teaching after PD with the support of PD (Guskey, 1994). In other words, it could take more than one workshop to provide teachers with the necessary tools to implement the strategies they had learned in PD (Birman et al., 2000). PD is a relatively long process. An effective PD program must be long enough to facilitate change (Birman et al., 2000; Guskey, 1994).

Time identifies both the hours scheduled during one session and a sustained focus stretched out over several sessions within the school year (Desimone, 2009). Sustained PD includes a series of sessions within a school year with a consistent feature of professional learning (Borko, 2004). In this way, teachers can internalize and accept an instructional approach/strategy from the PD in their daily teaching (Birman et al., 2000; Guskey, 2003). Time needs to be measured. As Fullan and Miles (1992) claimed, teachers need to obtain support from the presenters of the PD at least 30 days per year so that they can effectively implement what they learned in the PD.

On the other hand, researchers used training hours to show the time of PD instead of days and weeks to examine the effect of PD. For example, Blank et al. (2008) found that effective PDs were over 50 h by examining 25 teacher PDs. In addition, the real training hours are related to the effect of PD. Wayne et al. (2008) found that PDs with 30–100 training hours had the greatest impact on teachers’ knowledge and students’ performance. However, Desimone (2009) claimed a minimum requirement of 20 training hours for PD, and Supovitz and Turner (2000) suggested a minimum of 80 training hours for effective PD.

Generally, after experiencing a PD with a longer period (e.g., more than two years), teachers gradually improved their teaching (Johnson & Fargo, 2010). The factors of duration and training hours are two names of time. Therefore, the sustained and intensive feature of PD is the key to effective PDs. As Johnson and Fargo (2010) stated, the most effective PDs could last 30–100 training hours and spread over 6 to 12 months.

### 1.5. Self-Efficacy and Measurement of Self-Efficacy

Before Bandura’s (1997) definition of teacher self-efficacy, Berman et al. (1977) defined teacher self-efficacy as “the extent to which the teacher believes he or she has the capacity to affect student performance” (p. 137). Bandura (1997) stated that self-efficacy determines how people feel, think, motivate themselves, and behave. Self-efficacy has a significant effect on human accomplishment and emotional well-being. Similarly, teacher self-efficacy influences teaching practices and student performance. Based on this definition of self-efficacy, Tschannen-Moran and Hoy (2001) further clarified “given attainments” as desired outcomes of student engagement and learning. They proved that teacher self-efficacy had significant relationships with teachers’ persistence, enthusiasm, commitment, and instructional behavior, as well as student outcomes. In addition, Ainley and Carstens (2018) defined “given attainments” as students’ achievement and motivation. Therefore, there is a relationship between teacher self-efficacy and student performance and behaviors.

On the other hand, measuring teacher self-efficacy is a complicated process because the measurement depends on the definition of teacher self-efficacy. Many studies examined teacher self-efficacy by using teachers’ self-reports. However, it is essential to understand what the survey is measuring. All this influences the interpretation of the data and the implications of the analysis results. Although there are many surveys about teacher self-efficacy, two categories of surveys are widely used. First is the Science Teaching Efficacy Belief Instrument in Riggs and Enochs (1990). The scale consists of two subscales: (a) general efficacy and (b) personal efficacy. Later, it was modified to measure different subjects (i.e., mathematics (Enochs et al., 2000) and technology (Kelani, 2009)). Second, the Teachers’ Sense of Efficacy Scale from Tschannen-Moran and Hoy (2001) was developed based on Bandura’s Teacher Efficacy Scale. It consists of (a) efficacy for student engagement, (b) efficacy for instructional practices, and (c) efficacy for classroom management. Many researchers adapted TSES for mathematics teaching (Hamond, 2018; Lohman, 2019).

Besides the above two popular categories of surveys, Woolfolk and Hoy (1990) designed a Teacher Efficacy Scale, including Teacher Efficacy and Personal Efficacy. After 2000, some scholars revised the scale of self-efficacy based on Bandura’s scale. For example, S. Y. Yoon et al. (2014) developed a scale that includes four subscales. Pinner (2012) created the Teacher Self-Efficacy Retrospective Questionnaire. Carney et al. (2016) designed teachers’ self-efficacy regarding the levels of teacher preparedness, and Yu et al. (2023) developed a scale including three dimensions for Chinese secondary English teachers.

In sum, it is essential for researchers to understand what the scales of teacher self-efficacy are measuring so that researchers can design appropriate and responsive interventions for preservice and in-service teacher education.

### 1.6. Professional Development on Teacher Self-Efficacy

Teacher self-efficacy has been recognized as a significant factor in teaching quality (e.g., Lumpe et al., 2012). Teacher self-efficacy has become a focus of PD because it has been positively correlated with teaching effect, teacher emotion, student achievement, and student motivation (Tschannen-Moran & Hoy, 2001). According to Bandura’s theory of self-efficacy, teachers are motivated to perform teaching if they are confident in this teaching and believe that it will have a favorable result. Teachers with higher self-efficacy have a stronger commitment, and their students achieve higher performance (Ashton & Webb, 1986; Bandura, 1994). Therefore, PD focusing on the improvement of teacher self-efficacy has the potential to improve teaching practice and student outcomes.

Many teachers seek PD as a way to enhance their knowledge and skills and continue meeting their students’ needs. Previous research shows a positive effect between PD and teacher self-efficacy (DePiper et al., 2021; Kelley et al., 2020; Heppt et al., 2022). For instance, DePiper et al. (2021) found that the Visual Access to Mathematics professional development program had a positive impact on teachers’ self-efficacy in supporting English learners in math learning by employing the method of pre-/post-tests and control–experimental groups and recruiting 101 teachers from 47 schools. Additionally, Kelley et al. (2020) investigated the impact of a PD program (i.e., Teachers and Researchers Advancing Integrated Lessons in STEM) on high school teachers’ self-efficacy in integrated STEM instruction through a collaborative community of practice. They found that science teachers who participated in 70 h of PD over three years significantly increased their self-efficacy, while engineering technology teachers did not show a significant change in their self-efficacy. Similarly, Heppt et al. (2022) investigated the effectiveness of PD aimed at enhancing elementary school teachers’ language-support skills in science instruction. After conducting research for over two years in Germany, Heppt and her colleagues collected 32 teachers’ data and found that all teachers significantly improved their self-efficacy for teaching elementary school science after they attended a two-year PD intervention about developing language-support skills and pedagogical content knowledge in science.

### 1.7. The Educational Stage, Area, and PD’s Impact on Teachers’ Self-Efficacy

The grade level of teachers’ teaching is related to the content of PD. Elementary and secondary content with different emphases could require different formats of PD. Similarly, the same PD could have a different impact on the self-efficacy of teachers who are from different educational stages. Wu et al. (2024) reported that grade level significantly moderates PD effects—elementary-level PD tended to yield larger self-efficacy gains than PD targeting secondary teachers when they conducted a meta-analysis about the impact of STEM education on elementary and high school teachers’ self-efficacy. Wu et al. (2024) strongly support the grade-level moderator of PD. Similarly, Lee et al. (2013) conducted an empirical study about the relationship between teachers’ self-efficacy and pedagogical conceptual change by examining the change of 12 elementary teachers and 18 secondary teachers in self-efficacy and conceptual change. Lee and her colleagues found a significant difference in elementary and secondary teachers in teaching experience and self-efficacy after attending a drama-based instruction PD model, namely, elementary teachers had higher self-efficacy than secondary teachers. Therefore, the variable of educational stage is an important moderator.

In addition, the different cultures from the different areas could contribute to the variation in PD’s effect size. Gümüş and Bellibaş (2023) investigated the relationship between PD and teacher self-efficacy by analyzing teacher data from 32 countries and regions in TALIS 2013 data. They found that teachers from most countries had higher perception of self-efficacy had higher self-efficacy while teachers from Malaysia, Brazil, Norway, Abu Dhabi (UAE), Australia, Finland, Denmark, Portugal, Slovak Rep., and Korea. This study suggests that PD programs should be tailored to the specific contexts and needs of teachers in different countries and cultures. The area where the study has been completed could be a moderator of PD’s impact.

With the development of technology in education, the teaching environment has been changing, especially during the COVID-19 period. The PDs for supporting teachers’ integration of technology into their teaching to meet the requirements of the new teaching environment are needed. Many teachers have very low self-efficacy to adopt the new teaching requirements (Horvitz et al., 2015; Ma et al., 2021). In the next few years, the need for PD will be very strong. Educators need to know the characteristics of effective PD so that they can design an appropriate PD to support teachers’ real needs. However, the picture is still unclear. Meta-analyses focusing on the relationship between teacher self-efficacy and teacher PD are limited. In addition, a limited meta-analysis included the variable of time data in the analysis of the effect of PDs on self-efficacy. It is essential to summarize this relationship between the characteristics of PD and teacher self-efficacy during the past several decades and identify the main characteristics of effective PD.

### 1.8. Research Questions

The current study aims to measure the effectiveness of PDs focusing on in-service STEM teacher self-efficacy and analyze the effect of the characteristics of PDs on the effectiveness of PDs by moderator analysis. We identified 18 primary studies focusing on improving in-service STEM teachers’ self-efficacy and 19 effect sizes. Also, we evaluated related factors that moderate their impacts on self-efficacy. Therefore, the following research questions are addressed.

(1) What is the overall effect size of PD on STEM teachers’ self-efficacy?
(2) Which moderators of the characteristics of PD have an impact on the improvement of STEM teachers’ self-efficacy? In the present study, the moderators consisted of publication type, area, educational stage, PD format, PD content, participant size, duration, and training hour.
(3) Are there any differences in the effectiveness of PD with different scales of self-efficacy on STEM teachers’ self-efficacy?

## 2. Methods

### 2.1. Study Inclusion and Exclusion Criteria

To select samples and answer the research questions, the current study sets up the following six criteria. All the selected papers must meet all six criteria. (1) Studies must be empirical research focusing on the effects of PD on teacher self-efficacy. (2) Studies must be published or reported in English before 3 February 2024. (3) Studies must focus on in-service teachers in grades K-12, including science, technology, mathematics, engineering, or STEM teachers. However, studies focusing on learning disabilities or on students with social or emotional disorders were excluded. (4) Studies must include the measurement of the effect of PD on teacher self-efficacy. (5) Studies must have used a valid control group. And (6) studies must include the necessary information for the calculation of effect sizes.

### 2.2. Study Search

**Figure 1:**
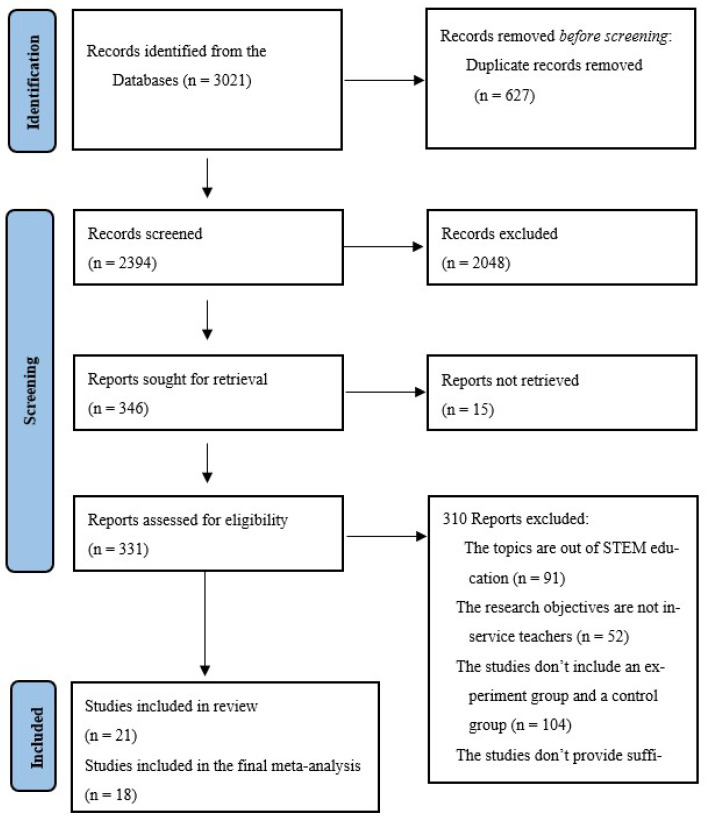
Literature search process.

This meta-analysis was prospectively registered on OSF at https://osf.io/b23rt (accessed on 22 July 2025). Based on the Preferred Reporting Items for Systematic Reviews and Meta-Analyses (PRISMA) (Table S1) guidelines and flow chart (Page et al., 2021), we reported this meta-analysis. We selected relevant studies published before 3 February 2024 by searching EBSCO host (ERIC, PsycINFO, Academic Search Premier, Teacher Reference Center), Web of Science, and ProQuest Dissertations & Theses Global (see Table S2). For example, the complete search used for Web of Science was: AB = (“professional development” OR “faculty development” OR “Staff development” OR “professional learning” OR “teacher training” OR “teacher improvement” OR “in-service teacher education” OR “peer coaching” OR “teacher’ institute*” OR “teacher mentoring” OR “Beginning teacher induction” OR “teachers’ Seminar*” OR “teachers’ workshop*” OR “teacher workshop*” OR “teacher center*” OR “teacher mentoring”) AND AB = (“teacher efficacy” OR “teaching efficacy”) AND AB = (“Math*” OR “Algebra*” OR “Number concepts” OR “Arithmetic” OR “Computation” OR “Data analysis” OR “Data processing” OR “Functions” OR “Calculus” OR “Geometry” OR “Graphing” OR “graphical displays” OR “graphic methods” OR “Science*” OR “Data Interpretation” OR “Laboratory Experiments” OR “Laboratory Procedures” OR “Experiment*” OR “Inquiry” OR “Questioning” OR “investigation*” OR “evaluation methods” OR “laboratories” OR “biology” OR “observation” OR “physics” OR “chemistry” OR “scientific literacy” OR “scientific knowledge” OR “empirical methods” OR “reasoning” OR “hypothesis testing” OR “engineering” OR “technology” OR “STEM”). We conducted a manual search using the reference lists of key articles published in English. The detailed search procedure is shown in Figure 1. After filtering all studies that do not fit the requirements in Figure 1, we found 21 studies for review, and finally, 18 were selected from the 21 studies because of outliers.

To analyze the characteristics of PD and teacher self-efficacy, we created a coding system (see Table 1) based on the above literature review. The coding framework includes two dimensions: Study and Intervention. Study includes publication, area, educational stage, research design, and instruments. Intervention includes PD format, PD content, duration, and training hours.

### 2.3. Study Coding

To ensure reliability, two coders separately coded all the studies based on the coding table. Then, we compared and discussed the coding until mutual agreement was reached (Burla et al., 2008). The initial agreement between the two coders was 92.1%. The disagreement was resolved after discussion.

### 2.4. Effect Size Calculation

In the current study, we used Hedges’*g* as the measure of effect size. Hedges’ *g* is a measure of effect size that eliminates the systematic bias that arises when the group sample sizes are small (i.e., *n* < 40; Glass et al., 1981). We calculated Hedges’*g* based on the formula of pre-post change in the treatment minus the mean pre-post change in the control group, divided by the pooled pretest standard deviation (Borenstein et al., 2011; Hedges, 2007; Morris, 2008).

A positive *g* would indicate that a PD has been effective in improving teachers’ self-efficacy. In cases where only inferential test results were reported (i.e., with means and standard deviations missing), *g* was estimated based on the inferential statistics, such as *t*, *F*, or *p* values (Wilson & Lipsey, 2001).

Given the wide-ranging forms of self-efficacy surveys (e.g., Section 1.5), we took a broad approach to the self-efficacy instruments employed in the primary studies. Specifically, we classified the self-efficacy instruments into three broad categories: (1) Teaching Efficacy Belief Instrument (TEBI), (2) Teacher’s Efficacy Beliefs Inventory (TSES), and (3) other survey instruments. Under this approach, each individual study was identified with one of these three types of self-efficacy instruments and was coded as such in our coding sheet.

### 2.5. Modeling Strategy

We used the Comprehensive Meta-Analysis (version 3.0) software for all statistical analyses. First, we identified whether there were any outliers. Second, we checked publication bias. Third, we modeled the overall effect size under a random-effects model (Cooper, 2010). Finally, we conducted moderator analyses when the groups of effect sizes had a high degree of heterogeneity (Cooper et al., 2019).

### 2.6. Outlier Control

We adopted influence analyses to detect potential outliers (Viechtbauer & Cheung, 2010). In this approach, the standardized deleted residuals were calculated first. If the standardized deleted residuals are out of the range of [−1.96, 1.96], then they are considered as outliers.

### 2.7. Publication Bias

To examine the possibility of publication bias, we first used a funnel plot to visually inspect the presence of publication bias. Asymmetries on either side of the funnel plot can indicate the presence of publication bias. Also, we employed Egger’s regression (Egger et al., 1997) to check the publication bias. Finally, we employed the trim-and-fill procedure to see if any adjustment for publication bias might be required (Duval & Tweedie, 2000). Furthermore, we analyzed the differences in effect sizes of PD between two kinds of studies (i.e., journal or non-journal). The results show that there are no significant differences in PD’s effect on self-efficacy between journal and non-journal studies.

### 2.8. Moderator Analysis

We used the Cochran *Q* test to determine heterogeneity between studies, and *I*^2^ to identify the magnitude of the heterogeneity between studies. Meanwhile, we selected a value of greater than 50% as the index of moderate-to-high heterogeneity (Higgins et al., 2003). In the current study, we focused on ten selected moderators based on our literature review (e.g., Section 1.1, Section 1.2, Section 1.3, Section 1.4, Section 1.5, Section 1.6 and Section 1.7). The purpose was to examine whether these moderators were significantly associated with the effects of PD on STEM teacher self-efficacy.

## 3. Results

### 3.1. Selected Studies

**Figure 2:**
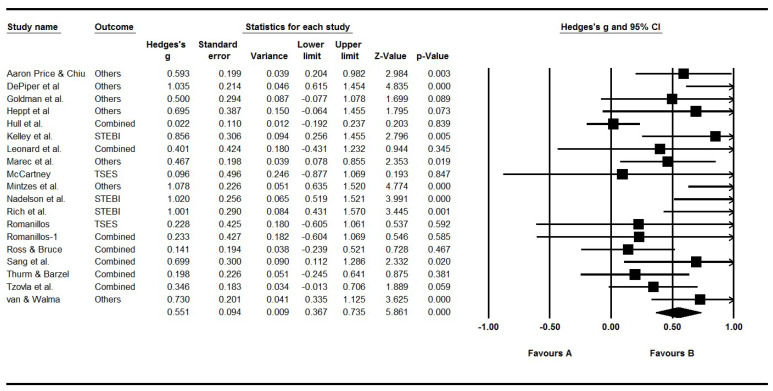
The forest plot of effect size. Note. Combined means we used the mean of multiple outcomes in the studies. Romanilos-1 means that the same article with different effect size. Articles included in the figure are: (Aaron Price & Chiu, 2018; DePiper et al., 2021; Goldman et al., 2019; Heppt et al., 2022; Hull et al., 2016; Kelley et al., 2020; Leonard et al., 2018; Marec et al., 2021; McCartney, 2013; Mintzes et al., 2013; Nadelson et al., 2013; Rich et al., 2017; Romanillos, 2017; Ross & Bruce, 2007; Sang et al., 2012; Thurm & Barzel, 2020; Tzovla et al., 2021; van Aalderen-Smeets & Walma van der Molen, 2015).

We identified 3021 studies. Ultimately, we identified 21 studies from 2007 to 2024 and calculated 23 effect sizes. Before analyzing the final data, we used influence analyses detect potential outliers (Viechtbauer & Cheung, 2010) and detected four extreme outliers which exceeded the SDRs limit ((Hopkins, 2018), *g* = −0.121, (Kaschalk-Woods et al., 2021), *g* = 3.679; (Rich et al., 2017), *g* = 1.607; (Trimmell, 2015), *g* = 2.504, see Table 2). Finally, we excluded three studies with very large effect sizes and one with a negative effect size, and then further analyzed 18 studies with 19 effect sizes in the current analysis (see Figure 2). The number of independent samples in each study, except Romanillos (2017), is one. The detailed information about included studies is shown in Table 2.

The current study assessed PD’s overall effect on STEM teacher self-efficacy and how PD’s effectiveness differed by the moderators of publication type, area, educational stage, PD format, PD content, duration, and training hours. Finally, we explored the effects of PD on different scales of teacher self-efficacy.

### 3.2. Overall Effectiveness of PD on STEM Teacher Self-Efficacy

To examine the overall effect of PD on STEM teachers’ self-efficacy, we conducted meta-analyses on the data set by weighting all effect sizes. We found that the mean effect sizes under a random-effects model were 0.551 (95% CI [0.367, 0.735], *p* < 0.001) (see Figure 2) and significantly different from zero (see Table 3).

### 3.3. Heterogeneity

The Q statistics show a significant result (Q_t_ (18) = 49.46, *p* < 0.001), which implies that the effect of PD on STEM teacher self-efficacy varied across studies. The Tau-squared value was 0.096, which suggests a meaningful distribution in the individual-study effect sizes across studies. Similarly, the I^2^ statistics showed that 63.61% of the observed heterogeneity could be accounted for by the variability between studies. These findings further confirm the necessity of moderator analyses. Meanwhile, some other factors, like the selected moderators, might play a role in creating variability.

### 3.4. Examining Publication Bias

**Figure 3:**
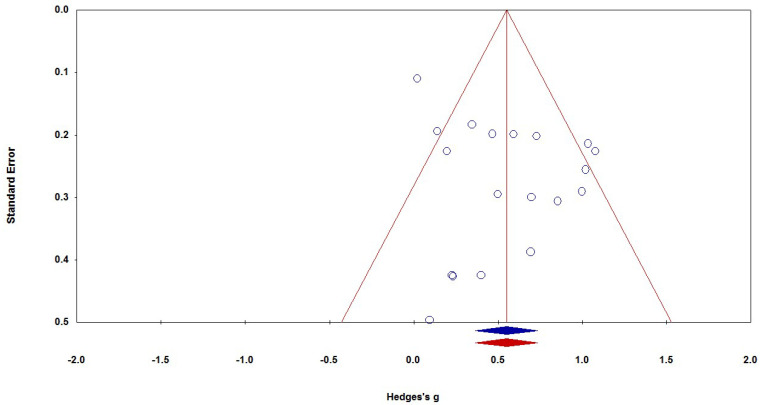
The funnel plot of effect size. Note. Each open circle represents an individual study’s effect size (Hedges’s g) plotted against its standard error. The red vertical line indicates the overall pooled effect size estimated from the random-effects model. The two red diagonal lines represent the 95% confidence limits around the summary effect, forming the expected “funnel” shape in the absence of publication bias. The blue and red rhombuses at the bottom represent the overall mean effect size before (red) and after (blue) adjustment for publication bias using the trim-and-fill method. Symmetry in the funnel suggests low publication bias.

In the current study, a funnel plot and Egger’s regression (Egger et al., 1997) were used to detect publication bias. The funnel plot shows that the 19 standard errors were relatively symmetrically distributed on both sides of the average effect size (see Figure 3). In addition, Egger’s regression (*t* (17) = 1.927, *p* = 0.071) shows no significant bias. Furthermore, we employed the trim-and-fill procedure to check the possible publication bias (Duval & Tweedie, 2000). The result shows that no studies on both sides of the distribution might have been missing under the random-effects model. Therefore, there was no evidence supporting that publication bias affected the estimated average effect size.

### 3.5. Moderator Analysis on the Overall Effect Sizes

We selected these nine variables for two reasons. First, these variables represent the characteristics of PD or research methodology. Second, at least two effect sizes are associated with each of the categories of the variable in the data set. Thus, we can further analyze the data. We explored nine variables that possibly have an impact on the effects of STEM teachers’ PD by conducting moderator analyses following the recommendations of Berlin and Antman (1994). We only provide the result of our focal moderator. The detailed results are shown in the following (see Table 4 and Table 5).

Publication type. The results show that journal papers reported statistically significant effects of PDs on self-efficacy (*g* = 0.586, *p* = 0.000). Despite a relatively small effect size (*g* = 0.192, *p* = 0.543), the PD effects in non-journal studies did not reach statistical significance. Moreover, the moderation analysis showed that there were no significant differences between the estimates of the average effects of journal papers and non-journal papers (*Q*_b_ (1) = 1.433, *p* = 0.231).

Area. The moderation analysis showed that the estimate of the average effects from the studies where the samples were from the USA (*g* = 0.750, *p* = 0.000) and non-USA (*g* = 0.347, *p* = 0.001) are significant, separately. In addition, the difference in effect sizes of PD between them is significant (*Q*_b_ (1) = 7.657, *p* = 0.006).

Educational stage. The moderation analysis showed that the estimated average effect size depended on the types of teachers. PDs focusing on primary teachers had the highest significantly estimated average effect size on self-efficacy (*g* = 0.607, *p* = 0.000). PDs focusing on secondary teachers had a significantly average effect size (*g* = 0.473, *p* = 0.005). However, PDs focusing on mixed teachers had a non-significantly positive effect size on self-efficacy (*g* = 0.526, *p* = 0.101). However, the effect sizes between any two categories of educational stage are not significant (*Q*_b_ (2) = 0.392, *p* = 0.822).

PD format. The moderation analysis showed that PDs with a non-traditional format have a highly significant effect on self-efficacy (*g* = 0.820, *p* = 0.000) and PDs with a traditional format have a modestly significant effect on self-efficacy (*g* = 0.462, *p* = 0.000). Furthermore, the moderation analysis showed that the difference in effect sizes between PDs with a traditional format and PDs with a non-traditional format is not significant (*Q*_b_ (1) = 3.250, *p* = 0.071).

PD content. The results showed that both the estimated average effect sizes of PD focusing on multidisciplinary content and science were significantly large and positive, separately (*g* = 0.608, *p* = 0.000; *g* = 0.731, *p* = 0.000). Meanwhile, it showed effect sizes of PDs focusing on mathematics were significant (*g* = 0.296, *p* = 0.040). However, the result of the comparison shows that the difference in effect sizes between PD focusing on science and PD focusing on mathematics was non-significant (*Q_b_* (1) = 3.31, *p* = 0.069).

Instruments of self-efficacy. The moderation analysis showed that the estimated average effect sizes of PD with TEBI and PD with other surveys were large significantly positive, separately (*g* = 0.684, *p* = 0.000; *g* = 0.654, *p* = 0.000); while the estimated average effect sizes of PD with TSES was small and non-significant (*g* = 0.080, *p* = 0.537 (see Table 5). Furthermore, the results show that the effect size of PD with TEBI was significantly higher than that of PD with TSES (*Q_b_* (1) = 17.175, *p* = 0.000), and the effect size of PD with other surveys was significantly higher than that of PD with TSES (*Q_b_* (1) = 16.215, *p* = 0.000).

Participant size, training hours, and duration of PD. We investigated whether the three continuous factors of duration, training hours, and participant size affected the estimate of the average effect of PD on teachers’ self-efficacy by using a meta-regression with a computational method of maximum likelihood. We first ran the meta-regression with each factor separately. The results showed that the factors of participant size (*B* = −0.0037, *p* = 0.016) and training hours (*B* = 0.0042, *p* = 0.047) were significant predictors for the estimated average effect of PD on STEM teachers’ self-efficacy, individually. However, duration was not a significant predictor for the estimated average effect of PD (*B* = 0.0050, *p* = 0.100, see Table 5). Furthermore, when controlling for the variable of training hours, the contribution of the factor of participant size to the effect size of PD on self-efficacy is significant (see Model 4: *B* = −0.0038, *p* = 0.001). It means that when PDs have the same training hours, the same number of participants increases by one unit, and the effect size of PD on self-efficacy decreases by 0.0038. Also, when controlling the two variables of duration and training hours, the factor of participant size is a significant predictor for the effect size of PD on self-efficacy (see Model 7: *B* = −0.0039, *p* = 0.001). It means that when PDs have the same duration and training hours, the same size increases by one unit, and the effect size of PD on self-efficacy decreases by 0.0039. Meta-regressions showed that the participant size in PD was significantly negatively associated with the effects of PD on teachers’ self-efficacy, but the factor of training hours in PD was significantly positively associated with the effects of PD on teachers’ self-efficacy. Generally, both of the two variables of participant size and training hours can significantly contribute to the effects of PD on teachers’ self-efficacy, while the variable of duration is not a significant contributor.

## 4. Discussion

### 4.1. Overall Effects of PD

The overall significant effect size of PDs on STEM in-service teachers’ self-efficacy under the random model was 0.551, which would be considered a medium effect size (Cohen, 1988). This result means that PD is an effective means of improving STEM in-service teachers’ self-efficacy, which is a key predictor of teaching effect. This finding is aligned with the results from Gesel et al. (2020) and Zhou et al. (2023). In-service PDs have significant effects on STEM teachers’ self-efficacy.

### 4.2. Effect Moderator of PD’s Format and Content

We found that PD programs with a non-traditional format make a greater contribution to the improvement of teachers’ self-efficacy than do PD programs that mainly use the mentoring format or lecturing format, while the difference was not significant. This finding is different from that of Egert et al. (2018). They found that PDs using solely coaching were three times more effective in PD quality rating than other programs. In addition, Egert et al. (2018) did not find significant differences in the effects on quality ratings and child outcome between PD programs with multiple delivery formats and PD programs using a single strategy (Egert et al., 2018). Similarly, we found a non-significant difference in the effect sizes between PD with a traditional lecturing format and PD with a mentoring and coaching format.

Our findings do support the suggestion from K. S. Yoon et al. (2007) that PD uses a non-traditional format to train in-service teachers rather than using only lecturing or mentoring. Yoon and his colleague encourage trainers to deliver theoretical knowledge through courses, workshops, or meetings and guide in-service teachers to practice when they learn in the PD program (K. S. Yoon et al., 2007). However, in the current study, we coded the formats into two general categories rather than into several subcategories that are more detailed. Because of the complicated formats of PD, future studies focusing on the relationship between more detailed PD formats and effect sizes are needed.

PD could have different emphasized content for participants. The findings from the moderator analysis of PD content reveal that PDs focusing on multidisciplinary content and science have a significant medium effect on self-efficacy, while PDs focusing on mathematics content have a small effect size on self-efficacy. However, the differences in effect sizes of PD on teachers’ self-efficacy are not significant. These findings do not support the results from Kennedy (1998). Kennedy (1998) found significant differences in effect sizes of PDs among the PDs focusing on math, science, and multidisciplinary content, and non-significant differences in the effect sizes of PDs among PDs with different forms and structures. Similarly, our findings do not support the results in Kraft et al. (2018). Kraft et al. (2018) found that coaching model PDs had a significant effect size on students’ math achievement rather than science. Based on our findings, we suggest that PDs could focus more on the understanding of math content to improve in-service teachers’ self-efficacy in math teaching. Math teachers might need more professional development programs, and the quality of math teachers’ PD programs might need to be improved. Teacher educators can use the identified features of PD to perfect their design of PDs. The different subjects’ PD designers can further learn from each other in the fast development of STEM education because PDs focusing on multidisciplinary content have a significant effect on in-service STEM teachers’ self-efficacy. Finally, mathematics education researchers could pay more attention to math teachers’ PD because the effect size of PD on math teachers’ self-efficacy is relatively low, and mathematics is one of the foundational subjects for STEM education.

### 4.3. Effect Moderators of PD’s Participant Size, Training Hours, and Duration

First, as many studies suggested, participant size is a significant predictor of the effect size of PD on teachers’ self-efficacy. Our findings further confirm this relationship. The factor of participant size is a significant factor that contributes to the change in the effect of PD on teachers’ self-efficacy. We found that there was a significantly negative relationship between the effect sizes of PD and participant size (see Model 1 in Table 5). However, this finding is different from that of Egert et al. (2018) because their findings do not support the notion that a large-scale PD is less effective than a small-scale PD. An appropriate size of PD can make the management of PD easy and enable more interaction between the trainer and trainee. Also, participants could have more time to communicate with others. As Hamre and Hatfield (2012) stated, PD programs might be most efficient when the number of participants is fewer than 30, and a larger or smaller size may be needed only when the focus of PD is general and broad or more detailed and involving deep learning. The reasons for this need to be explored further in the future.

Second, the factor of training hours was significantly associated with the effects of PD programs on teacher self-efficacy. Training hours can increase the effect of PD on teachers’ self-efficacy based on our findings. This aligns with Johnson and Fargo (2010). However, our finding is different from Kalinowski et al. (2020). They found there was no significant relationship between the effect sizes of PD and time. However, Werner et al. (2016) found a curvilinear relationship between training hours and the effects of PD, but they used 10 training hours as the bar. Like Hamre and Hatfield (2012), when these PDs target specific skills, short-term programs might be sufficient; when the focus of PDs is comprehensive and broad, long-term, and intensive PD may be needed. Similarly, Basma and Savage (2018) showed that PDs with fewer than 30 h rather than more than 30 h led to higher student literacy. Their findings support the notion that fewer PD training hours produce higher quality than do PDs with longer training hours. The real reasons for these results still need to be explored in the future.

Next, our findings do not support the positive significant relationship between the duration of PD and the effect of PDs. It suggests that PD designers need to find an appropriate duration rather than a longer period. The reasons could be that participants felt overfatigued. However, the exploration of the real reasons is still needed in the future. For example, Zhou et al. (2023) found that PD duration significantly increased their self-efficacy for teachers who received STEM-focused pedagogy training, compared to those who did not. Furthermore, the studies including duration of PD and training hours are limited, and the difference in the definitions between duration of PD and training hours is significant. Therefore, with more PD using the mixed format, including traditional and non-traditional, the exploration of the difference in effect sizes between duration and training hours is needed.

Finally, we found that model 4 was the best fit for the data. Model 4 includes two factors of participant size and training hours. Both factors are significant contributors to effect sizes. It suggests that PD designers could consider the two significant factors when designing their PDs: appropriate participant size and training hours. As Hamre and Hatfield (2012) stated, PD programs might be most efficient when the number of participants is fewer than 30, and a larger or smaller size may be needed only when the focus of PD is general and broad or more detailed and involving deep learning. Similarly, Egert et al. (2018) found that PDs with 45–60 training hours appeared to be most effective in improving PDs’ effects on external rating as compared to PDs with both shorter and longer training hours.

### 4.4. Effect Moderator of Educational Stages and Areas

We found that PDs targeting primary teachers and secondary teachers had significant effects on self-efficacy, but the difference in effect size of PDs is not significant. This is different from Egert et al. (2018). As Egert et al. (2018) claimed, primary teachers attending the PD program showed more improvement in self-efficacy than did secondary teachers. This may indicate that primary teachers’ PD is adaptive to the learning needs of elementary teachers and their professional context (Buysse et al., 2009; Egert et al., 2018). Primary teachers and secondary teachers obviously have different professional backgrounds. The requirements of content knowledge for secondary teachers may be higher than for elementary teachers. However, the characteristics of PD trainers—such as background, experience, profession, and qualification—cannot be ignored when one is analyzing the effect of PD (Egert et al., 2018). We suggest that supplements should include information about the PD procedure and trainers’ backgrounds. Additionally, we found that the effect of PD focusing on mixed-grade-level teachers was non-significant. Although PDs targeting mixed-grade-level teachers could provide many opportunities for them to communicate teaching coherence across grade levels, the big differences in the content knowledge between primary and secondary levels could block them from communicating deeply. Therefore, the reasons for the differences in the effect sizes of PD between primary teachers and secondary teachers require further study.

On the other hand, our findings supported that the USA PDs had a significantly higher estimated average effect on self-efficacy. Findings regarding the location of PD are still needed to confirm further. The reasons are complicated. This may be related to the fact that the USA has more educational published studies than others do.

### 4.5. Diversity of Self-Efficacy Tools

The validity of self-efficacy tools used in the studies is the key to the effect size of PD on self-efficacy. The between-study heterogeneity might be the result of the use of the scales of self-efficacy without psychometric support and could weaken the conclusion of this meta-analysis. Furthermore, it is essential for researchers to ensure the reliability and validity of the scale of self-efficacy. When using the scales without psychometric support, conclusions about the effects of PDs on teacher self-efficacy might not be reliable. More studies may need to evaluate the scales of self-efficacy, as small differences in the conceptualization and measurement of self-efficacy may influence the effects of PD on self-efficacy.

The results show that PDs using the TEBI survey had a significant effect on self-efficacy, while PDs using another popular TSES survey did not have a significant effect on self-efficacy. This is different from Chesnut and Burley (2015). They found that the measurement of self-efficacy based on a more accurate conception contributed to the higher effect sizes. In the current study, we assumed that TEBI and TSES were accurate compared to others, but we did not find a significant effect of PDs using TSES on self-efficacy. Meanwhile, we found the effect size of PD using other surveys is significant, and the differences in effect sizes between PD using TEBI and TSES are significant. It does not mean that the reliability and validity of TSES are poorer than TEBI. Future research may need to further examine the contributions of the types of self-efficacy. For example, the TEBI scale includes two subcategories: Personal Science Teaching Efficacy and Science Teaching Outcome Expectancy. This might be related to the conceptualization of the TEBI scale of self-efficacy. The second subcategory is to examine teachers’ expectations about their real teaching effects after their self-efficacy improves, while the first subcategory is to examine their self-reported confidence in teaching. However, TSES focuses more on teachers’ self-reported beliefs in their teaching. It includes self-efficacy in student engagement, self-efficacy in instructional practices, and self-efficacy in classroom management. The survey based on different definitions of self-efficacy might explain the inconsistent findings about the effects of PDs on self-efficacy. More studies are needed to explore the reliability of self-efficacy scales.

In sum, although many studies have suggested a directional effect of PD on self-efficacy (Ross & Bruce, 2007; Ribeiro, 2009; Lohman, 2019), in the current meta-analysis, we did not attempt to test the causal effect of this relationship. However, the directional medium effect of PD on STEM teachers’ self-efficacy indicates that teachers have higher self-efficacy after they attend PDs.

### 4.6. Limitations and Future Research

There are several limitations to this study. First, we selected studies that focus on in-service PD involving teacher self-efficacy. It is necessary to analyze the effect of pre-service teachers’ PD on self-efficacy in the near future. Second, the majority of selected studies were in the USA. To extend the evidence, experimental studies in other countries must be identified. The findings in this study must be discussed under the requirements of teacher education in the USA. A potential third limitation is that we explored general teachers’ self-efficacy rather than a special type of self-efficacy, like self-efficacy in instruction, or others. Although the selected studies employed different scales of self-efficacy, we further coded them into three categories so that we could employ moderator analyses to analyze the effects of the different scales of self-efficacy. It is essential to explore whether the reliability of the scale of self-efficacy has a significant impact on the effect sizes of PD in the future. Fourth, this meta-analysis examines the relationship between PD with different characteristics and STEM teachers’ self-efficacy rather than confirming causality. Thus, we must emphasize that no causality could be drawn from this meta-analysis. Finally, there was significant heterogeneity across studies. Although we conducted several moderate analyses, we did not conduct the interaction effects of moderators, like the interaction of format and duration. Future studies can use Meta-CART (X. Li et al., 2020) to examine whether the interaction effects of the characteristics of PD have significant impacts on the effect sizes of PD.

## 5. Conclusions

This meta-analysis synthesized studies focusing on the relative effectiveness of PD on K-12 in-service STEM teachers’ self-efficacy. In addition, it provides meta-analytic evidence that PD contributes to the improvement of STEM teachers’ self-efficacy. The findings confirm the significant relationship between STEM teachers’ PD and teacher self-efficacy by showing that the overall PD has a modest, significantly positive impact on K-12 STEM teachers’ efficacy. Based on the moderator analyses, we have several significant findings. The first is that the effect of PD having more training hours has a higher effect size. The second is that the effect of PD having fewer participants has a higher effect size. Third, we found the differences in effect sizes of PDs using different scales of self-efficacy. PDs using TEBI had a significant effect size. Therefore, we suggest that PD designers consider participant size, PD training hours, and the survey of self-efficacy when they hold PD programs. Also, for the measurement of the effect of PDs, we suggest that researchers consider the two aspects: the survey of self-efficacy and control groups of PDs. The findings from this meta-analysis could provide a broader picture of effective PDs for teacher self-efficacy for teacher educators, as well as offering possible solutions for developing effective PDs to enhance STEM teachers’ self-efficacy.

## Figures and Tables

**Table 1:** The description of codes.

Code	Description
Study	
Publication type	(1) Peer-reviewed journal, (2) non-journal (chapter, conference, dissertation or thesis, report, other).
Area	(1) USA, (2) other countries.
Educational stage	(1) Elementary (K-5), (2) secondary (G 6–12), (3) mixed (K-12).
Instruments	The scale of teacher self-efficacy * (1) Science Teaching Efficacy Belief Instrument (STEBI), (2) Teacher’s Efficacy Beliefs Inventory (TSES), (3) others.
Participant size	Number of teachers participating in the study.
Intervention	
Format	Delivery format(s) employed. (1) Tradition (include workshops, courses, and conferences); (2) non-tradition (include tradition and study group, or mentoring, or coaching).
Content	(1) Mathematics, (2) science, (3), technology, (4) engineering, (5) multidiscipline.
Duration time	Total duration in months that the PD lasts. If a study did not include the information about duration in months, we calculated it in terms of months. For example, one school year equals nine months.
Training hour	Training of PD in hours. If a study did not show PD training time in hours, we calculated the training time by using eight hours instead of one full day.
Effect size level	
Statistical data	Outcome data for meta-analysis. We used sample sizes, means, standard deviations, pre-post correlations, t, p, F, d to calculate effect size.

Note *. There are more than 10 different scales of self-efficacy appeared in the studies: (a) STEBI, (b) TSES, (c) MSES, (d) DAS-TE, (e) T-STEM, (f) TSI, (g) CRTSE/CRTOE, (h) any other modified or edited scales by researchers and based on Bandura’s theory. According to the literature review about self-efficacy, the scale of self-efficacy from Riggs and Enochs (1990) has been revised and developed in the past several decades after many empirical examinations. We grouped the first scales into the category of STEBI (1) and kept the second and third scales in the category of TSES (2). Finally, the rest of the scales were classified into the category of others (3).

**Table 2:** Characteristics of included studies.

Study	Instruments	PD Format	PD Content	Duration (Week)	Training Hour	Education Stage	Publication Type	Area	Participant Size	ES (g)
Aaron Price and Chiu (2018)	DAS-TE ^(3)^	tradition	S	36	56	mixed	journal	USA	78	0.593
DePiper et al. (2021)	Author Modified ^(3)^	tradition	M	40	50	secondary	journal	USA	52	1.035
Goldman et al. (2019)	Author Modified ^(3)^	tradition	S	36	88	secondary	journal	USA	23	0.500
Heppt et al. (2022)	Author Modified ^(3)^	tradition	S	80	76	primary	journal	Germany	10	0.695
Hopkins (2018)	STEBI-PSTE/STOE ^(1)^	tradition	S	36	100	mixed	non-journal	USA	60	−0.121
Hull et al. (2016)	TSES-CM/IS/SE ^(2)^	tradition	M	36	34	primary	journal	Belize	166	0.022
Kaschalk-Woods et al. (2021)	STEBI PSTE/STOE ^(1)^	tradition	S	20	5	secondary	journal	USA	22	3.679
Kelley et al. (2020)	T-STEM ^(1)^	tradition	STEM	2	70	secondary	journal	USA	30	0.856
Leonard et al. (2018)	CRTSE/CRTOE ^(3)^	tradition	STEM	8	24	mixed	journal	USA	10	0.401
Marec et al. (2021)	DAS-TE ^(3)^	non-tradition	STEM	36	36	primary	journal	Canada	69	0.467
McCartney (2013)	MSES ^(2)^	tradition	M	4	8	primary	non-journal	USA	6	0.096
Mintzes et al. (2013)	TSI ^(3)^	non-tradition	S	108	170	primary	journal	USA	48	1.078
Nadelson et al. (2013)	STEBI ^(1)^	tradition	STEM	1	24	primary	journal	USA	36	1.020
Rich et al. (2017)	T-STEM ^(1)^	tradition	T	36	27	primary	journal	USA	27	1.001
Rich et al. (2017) (1)	T-STEM ^(1)^	tradition	E	36	27	primary	journal	USA	27	1.607
Romanillos (2017)	TSES ^(2)^	tradition	S	1	40	secondary	non-journal	USA	12	0.228
Romanillos (2017) (1)	STEBI-PSTE/STOE ^(1)^	tradition	S	1	40	secondary	non-journal	USA	12	0.233
Ross and Bruce (2007)	TSES-CM/IS/SE ^(2)^	tradition	M	2	14	secondary	journal	Canada	57	0.141
Sang et al. (2012)	STEBI-PSTE/STOE ^(1)^	non-tradition	STEM	10	10	primary	journal	China	23	0.699
Thurm and Barzel (2020)	Author Modified ^(3)^	tradition	M	24	24	secondary	journal	Germany	39	0.198
Trimmell (2015)	STEBI ^(1)^	tradition	STEM	72	50	primary	non-journal	USA	25	2.504
Tzovla et al. (2021)	STEBI ^(1)^	tradition	STEM	5	48	primary	Journal	Greece	127	0.346
van Aalderen-Smeets and Walma van der Molen (2015)	DAS-TE ^(3)^	tradition	S	24	18	primary	journal	Netherlands	61	0.730

Note. M = mathematics, S = science, STEM = multidisciplinary subject. STEBI = Science Teaching Efficacy Belief Instrument; PSTE = Personal Science Teaching Efficacy Belief; STOE = Science Teaching Outcome Expectancy; TSES = Teachers’ Sense of Efficacy Scale; MSES = Mathematics Self-Efficacy Scale; DAS-TE = Dimensions of Attitude towards Science-Self efficacy; TSI = Teaching Science as Inquiry; T-STEM = the Friday Institute for Educational Innovation’s Teacher Efficacy and Attitudes Toward STEM Survey; CRTSE = culturally responsive teaching self-efficacy, CRTOE = culturally responsive teaching outcome expectancy; Instruments: the scale of teacher self-efficacy ^(1)^ means STEBI category, ^(2)^ means TSES category, ^(3)^ means other. Rich et al. (2017) (1) and Romanillos (2017) (1) mean that the two studies has another effect size, separately.

**Table 3:** Overall effectiveness of PD on STEM teacher self-efficacy.

Model	K	Effect Size	95% CI	Test of Null	Heterogeneity		
		*g* (SE)		Z(*p*)	Q (df)	*p*	*I* ^2^
Random	19	0.551(0.094)	[0.367, 0.735]	5.860 (0.000)	49.46 (18)	0.000	63.61%

**Table 4:** The moderators on the overall effect sizes under random effects model.

Variable	*k*	*g*	SE	95% CI	*Z*	*p*	Q_b_	df	p_b_
Publication type							1.433	1	0.231
Journal	16	0.586	0.162	[0.392, 0.780]	5.925	0.000			
Non-journal	3	0.192	0.315	[−0.425, 0.808]	0.609	0.543			
Area							7.657	1	0.006
USA	11	0.750	0.106	[0.542, 0.958]	7.060	0.000			
Other	8	0.347	0.100	[0.151, 0.542]	3.471	0.001			
Educational Stage							0.392	2	0.822
Primary	10	0.607	0.135	[0.342, 0.873]	4.485	0.000			
Secondary	7	0.473	0.170	[0.140, 0.805]	2.785	0.005			
Mixed	2	0.526	0.321	[−0.103, 1.156]	1.639	0.101			
Format							3.250	1	0.071
Tradition	15	0.462	0.097	[0.272, 0.652]	4.762	0.000			
Non-tradition	4	0.820	0.173	[0.480, 1.160]	4.726	0.000			
Content							4.714	2	0.094
M	5	0.296	0.144	[0.013, 0.578]	2.053	0.040			
S	5	0.731	0.154	[0.430, 1.032]	4.754	0.000			
Multidiscipline	9	0.608	0.125	[0.362, 0.854]	4.848	0.000			
Instruments							15.505	2	0.000
STEBI	6	0.684	0.125	[0.440, 0.928]	5.489	0.000			
TSES	4	0.080	0.129	[−0.173, 0.332]	0.618	0.537			
Others	9	0.654	0.092	[0.473, 0.835]	7.076	0.000			
Instruments 1							17.175	1	0.000
STEBI	6	0.667	0.110	[0.451, 0.883]	6.047	0.000			
TSES	4	0.063	0.095	[−0.230, 0.954]	1.200	0.230			
Instruments 2							16.215	1	0.000
TSES	4	0.073	0.115	[−0.153, 0.300]	0.637	0.524			
Others	7	0.655	0.087	[0.485, 0.825]	7.536	0.000			

**Table 5:** Results of random-effects meta-regression analyses.

	*K*	*B*	*SE*	95% CI	*p*	R^2^ Analog
Model 1	19					0.55
Intercept		0.7666	0.1199	[0.5316, 1.0017]	0.0000	
Participant size		−0.0037	0.0015	[−0.0066, −0.0007]	0.0157	
Model 2						0.28
Intercept		0.3576	0.1226	[0.1173, 0.5980]	0.0035	
Training hour		0.0042	0.0021	[0.0001, 0.0083]	0.0471	
Model 3						0.17
Intercept		0.4069	0.1177	[0.1761, 0.6376]	0.0005	
Duration		0.0050	0.0030	[−0.0010, 0.0109]	0.1001	
Model 4						0.89
Intercept		0.6029	0.1293	[0.3495, 0.8563]	0.0000	
Participant size		−0.0038	0.0012	[−0.0061, −0.0015]	0.0010	
Training hour		0.0039	0.0016	[0.0008, 0.0071]	0.0154	
Model 5						0.27
Intercept		0.3563	0.1232	[0.1148, 0.5978]	0.0038	
Duration		0.0007	0.0050	[−0.0091, 0.0106]	0.8847	
Training hour		0.0037	0.0036	[−0.0033, 0.0108]	0.2988	
Model 6						0.80
Intercept		0.6478	0.1248	[0.4032, 0.8924]	0.0000	
Participant size		−0.0041	0.0013	[−0.0065, −0.0016]	0.0012	
Duration		0.0051	0.0024	[0.0005, 0.0098]	0.0305	
Model 7						0.88
Intercept		0.6057	0.1297	[0.3515, 0.8600]	0.0000	
Participant size		−0.0039	0.0012	[−0.0063, −0.0016]	0.0010	
Training hour		0.0028	0.0046	[−0.0027, 0.0084]	0.3187	
Duration		0.0018	0.0040	[−0.0060, 0.0097]	0.6429	

## Data Availability

The original contributions presented in this study are included in the article/Supplementary Material.

## Supplementary Materials

The following supporting information can be downloaded at: https://www.mdpi.com/article/10.3390/bs15101364/s1, Table S1: PRISMA 2020 Checklist; Table S2: Search Queries Used for Each Database; Table S3: Description of The Included Studies.
