# Supplementary material for: The Effectiveness of Professional Development in the Self-Efficacy of In-Service Teachers in STEM Education: A Meta-Analysis
Source: Behav Sci (Basel). 2025 Oct 6;15(10):1364. doi: 10.3390/bs15101364 (PMC12561411; doi:10.3390/bs15101364)
Supplement: Supplementary file 1 [file behavsci-15-01364-s001.zip › behavsci-3793154-supplementary.pdf]

Supplementary Table S1: *PRISMA 2020 Checklist*

Section and Topic	Item #	Checklist Item	Location in Manuscript
TITLE			
Title	1	Identify the report as a systematic review.	Title (Meta-analysis)
ABSTRACT			
Abstract	2	See the PRISMA 2020 for Abstracts checklist.	Abstract
INTRODUCTION			
Rationale	3	Describe the rationale for the review in the context of existing knowledge.	Introduction
Objectives	4	Provide an explicit statement of the objective(s) the review addresses.	Research Questions section (end of Introduction)
METHODS			
Eligibility criteria	5	Specify the inclusion and exclusion criteria and grouping.	Study Inclusion and Exclusion Criteria
Information sources	6	Specify all information sources used to identify studies.	Study Search section: ERIC, PsycINFO, ProQuest, Google Scholar
Search strategy	7	Present full search strategies for all databases used.	Study Search and Figure 1 (PRISMA diagram)
Selection process	8	Specify the methods used to decide on inclusion.	Study Inclusion and Exclusion Criteria
Data collection process	9	Specify methods used to collect data from reports.	Study Coding section
Data items	10	List and define all outcomes for which data were sought.	Study Coding and Coding Table 1
Study risk of bias assessment	11	Specify methods used to assess risk of bias.	Not reported (Limitation noted)
Effect measures	12	Specify effect measure(s) used for each outcome.	Effect Size Calculation section
Synthesis methods	13a–f	Describe synthesis methods and heterogeneity investigations.	Modeling Strategy, Meta-Regression, Moderator Analysis
Reporting bias assessment	14	Describe any methods used to assess reporting bias.	Publication Bias section
Certainty assessment	15	Describe methods to assess certainty in evidence.	Not reported (Limitation noted)
RESULTS			
Study selection	16	Describe search results and selection process.	Figure 1 and Study Search section

Study characteristics	17	Cite and present characteristics of each included study.	Table 2 and Study Coding section
Risk of bias in studies	18	Present risk of bias assessments for each study.	Not reported
Results of individual studies	19	Present summary data for all outcomes of each study.	Table 2, Results section
Results of syntheses	20	Present results of all statistical syntheses.	Results and Moderator Analysis sections
Reporting biases	21	Present assessments of reporting biases.	Publication Bias section
Certainty of evidence	22	Present assessments of certainty in evidence.	Not reported
DISCUSSION			
Discussion	23	Interpret results, discuss limitations, implications.	Discussion and Limitations sections
OTHER INFORMATION			
Registration and protocol	24	Provide registration info and access to protocol.	Study Search (OSF: Registration DOI https:// OSF.IO/B23RT)
Support	25	Describe financial or non-financial support.	Funding section
Competing interests	26	Declare any competing interests.	Conflicts of Interest section
Availability of data, code, and other materials	27	Report availability of review materials.	Data Availability section

Source: https://www.prisma-statement.org/prisma-2020-checklist

Supplementary Table S2: *Search Queries Used for Each Database*

Database	Query
**EBSCO** host (ERIC, PsycINFO, Academic Search Premier, Teacher Reference Center)	(“professional development” OR “faculty development” OR “Staff development” OR “professional learning” OR “teacher training” OR “teacher improvement” OR “in-service teacher education” OR “peer coaching” OR “teacher' institute*” OR “teacher mentoring” OR “Beginning teacher induction” OR “teachers' Seminar*” OR “teachers' workshop*” OR “teacher workshop*” OR “teacher center*” OR “teacher mentoring”) AND (“teacher efficacy” OR “teaching efficacy”) AND (“Math*” OR “Algebra*” OR “Number concepts” OR “Arithmetic” OR “Computation” OR “Data analysis” OR “Data processing” OR “Functions” OR “Calculus” OR “Geometry” OR “Graphing” OR “graphical displays” OR “graphic methods” OR “Science*” OR “Data Interpretation” OR “Laboratory Experiments” OR “Laboratory Procedures” OR “Experiment*” OR “Inquiry” OR “Questioning” OR “investigation*” OR “evaluation methods” OR “laboratories” OR “biology” OR “observation” OR “physics” OR “chemistry” OR “scientific literacy” OR “scientific knowledge” OR “empirical methods” OR “reasoning” OR “hypothesis testing” OR “engineering” OR “technology” OR “STEM”)
**Web of Science**	AB = (“professional development” OR “faculty development” OR “Staff development” OR “professional learning” OR “teacher training” OR “teacher improvement” OR “inservice teacher education” OR “peer coaching” OR “teacher' institute*” OR “teacher mentoring” OR “Beginning teacher induction” OR “teachers' Seminar*” OR “teachers' workshop*” OR “teacher workshop*” OR “teacher center*” OR “teacher mentoring”) AND AB = (“teacher efficacy” OR “teaching efficacy”) AND AB =(“Math*” OR “Algebra*” OR “Number concepts” OR “Arithmetic” OR “Computation” OR “Data analysis” OR “Data processing” OR “Functions” OR “Calculus” OR “Geometry” OR “Graphing” OR “graphical displays” OR “graphic methods” OR “Science*” OR “Data Interpretation” OR “Laboratory Experiments” OR “Laboratory Procedures” OR “Experiment*” OR “Inquiry” OR “Questioning” OR “investigation*” OR “evaluation methods” OR “laboratories” OR “biology” OR “observation” OR “physics” OR “chemistry” OR “scientific literacy” OR “scientific knowledge” OR “empirical methods” OR “reasoning” OR “hypothesis testing” OR “engineering” OR “technology” OR “STEM”

**ProQuest**	abstract("professional development" OR "faculty development" OR "Staff development" OR "professional learning" OR "teacher training" OR "teacher improvement" OR "in-service teacher education" OR "peer coaching" OR "teacher' institute*" OR "teacher mentoring" OR "Beginning teacher induction" OR "teachers' Seminar*" OR "teachers' workshop*" OR "teacher workshop*" OR "teacher center*" OR "teacher mentoring") AND abstract("teacher efficacy" OR "teaching efficacy") AND abstract("Math*" OR "Algebra*" OR "Number concepts" OR "Arithmetic" OR "Computation" OR "Data analysis" OR "Data processing" OR "Functions" OR "Calculus" OR "Geometry" OR "Graphing" OR "graphical displays" OR "graphic methods" OR "Science*" OR "Data Interpretation" OR "Laboratory Experiments" OR "Laboratory Procedures" OR "Experiment*" OR "Inquiry" OR "Questioning" OR "investigation*" OR "evaluation methods" OR "laboratories" OR "biology" OR "observation" OR "physics" OR "chemistry" OR "scientific literacy" OR "scientific knowledge" OR "empirical methods" OR "reasoning" OR "hypothesis testing" OR "engineering" OR "technology" OR "STEM")

Supplementary Table S3: *Description of the Included Studies*

Study	Instruments	PD format	PD content	Duration (Week)	Training hours	Education stage	Publication type	Area	participant size	Pre/post-test	ES (g)
Aaron Price & Chiu (2018)	DAS-TE (3)	tradition	S	36	56	mixed	journal	USA	78	yes	0.593
DePiper et al. (2021)	Author Modified (3)	tradition	M	40	50	secondary	journal	USA	52	yes	1.035
Goldman et al. (2019)	Author Modified (3)	tradition	S	36	88	secondary	journal	USA	23	no	0.500
Heppt et al. (2022)	Author Modified (3)	tradition	S	80	76	primary	journal	Germany	10	yes	0.695
Hopkins (2018)	*STEBI-PSTE/STOE (1)*	*tradition*	*S*	*36*	*100*	*mixed*	*non-journal*	*USA*	*60*	*no*	*-0.121*
Hull et al. (2016)	TSES-CM/IS/SE (2)	tradition	M	36	34	primary	journal	Belize	166	yes	0.022
Kaschalk-Woods et al. (2021)	*STEBI PSTE/STOE (1)*	*tradition*	*S*	*20*	*5*	*secondary*	*journal*	*USA*	*22*	*yes*	*3.679*
Kelley et al. (2020)	T-STEM (1)	tradition	STEM	2	70	secondary	journal	USA	30	no	0.856
Leonard et al. (2018)	CRTSE/CRTOE (3)	tradition	STEM	8	24	mixed	journal	USA	10	no	0.401
Marec et al. (2021)	DAS-TE (3)	non-tradition	STEM	36	36	primary	journal	Canada	69	yes	0.467
McCartney (2013)	MSES (2)	tradition	M	4	8	primary	non-journal	USA	6	yes	0.096
Mintzes et al. (2013)	TSI (3)	non-tradition	S	108	170	primary	journal	USA	48	yes	1.078
Nadelson et al. (2013)	STEBI (1)	tradition	STEM	1	24	primary	journal	USA	36	no	1.020
Rich et al. (2017)	T-STEM (1)	tradition	T	36	27	primary	journal	USA	27	no	1.001
Rich et al. (2017) (1)	*T-STEM (1)*	*tradition*	*E*	*36*	*27*	*primary*	*journal*	*USA*	*27*	*no*	*1.607*
Romanillos (2017)	TSES (2)	tradition	S	1	40	secondary	non-journal	USA	12	no	0.228
Romanillos (2017) (1)	STEBI-PSTE/STOE (1)	tradition	S	1	40	secondary	non-journal	USA	12	no	0.233
Ross & Bruce (2007)	TSES-CM/IS/SE (2)	tradition	M	2	14	secondary	journal	Canada	57	yes	0.141

Sang et al. (2012)	STEBI-PSTE/STOE (1)	non-tradition	STEM	10	10	primary	journal	China	23	yes	0.699
Thurm & Barzel (2020)	Author Modified (3)	tradition	M	24	24	secondary	journal	Germany	39	no	0.198
Trimmell (2015)	*STEBI (1)*	*tradition*	*STEM*	*72*	*50*	*primary*	*non-journal*	*USA*	*25*	*no*	*2.504*
Tzovla et al. (2021)	STEBI (1)	tradition	STEM	5	48	primary	Journal	Greece	127	yes	0.346
van Aalderen-Smeets & Walma van der Molen (2015)	DAS-TE (3)	tradition	S	24	18	primary	journal	Netherlands	61	yes	0.730
